# Predictive Validity of a New Instrumental Activities of Daily Living Scale for Detecting the Incidence of Functional Disability among Community-Dwelling Older Japanese Adults: A Prospective Cohort Study

**DOI:** 10.3390/ijerph17072291

**Published:** 2020-03-29

**Authors:** Keitaro Makino, Sangyoon Lee, Seongryu Bae, Yohei Shinkai, Ippei Chiba, Hiroyuki Shimada

**Affiliations:** Department of Preventive Gerontology, Center for Gerontology and Social Science, National Center for Geriatrics and Gerontology, Obu 474-8511, Japan; sylee@ncgg.go.jp (S.L.); bae-sr@ncgg.go.jp (S.B.); yshinkai@ncgg.go.jp (Y.S.); ichiba@ncgg.go.jp (I.C.); shimada@ncgg.go.jp (H.S.)

**Keywords:** activities of daily living, community dwelling, disability evaluation, elderly, prospective study, screening tool

## Abstract

We examined the predictive validity of a newly developed scale—the National Center for Geriatrics and Gerontology Activities of Daily Living (NCGG-ADL)—to measure instrumental activities of daily living (IADL) ability. We tested the scale for detecting new incidences of functional disability among community-dwelling older Japanese adults. Participants were 2708 older adults (mean age = 79.0 years, 51.6% women) living in the community who had no functional decline at baseline. We assessed IADL ability using the NCGG-ADL scale, comprising 13 self-report questions. Next, we assessed their functional disability monthly for 24 months, based on the national long-term care insurance (LTCI) system. Among all participants, 430 (15.9%) had an IADL limitation at baseline, and 289 (10.7%) were newly certified as functionally disabled. Participants scoring ≤ 12 of 13 points in the NCGG-ADL showed a significantly higher risk of functional disability than did those scoring 13 points, even after adjusting for covariates (hazard ratio [95% confidence interval] = 1.58 [1.19–2.09]). We thus validated the NCGG-ADL as a screening tool for assessing the risk of functional disability among community-dwelling older Japanese adults. We conclude that IADL limitations, as measured by the NCGG-ADL, could be useful predictors of functional disability.

## 1. Introduction

At present, Japan is facing a population decrease and is becoming a super-aged society. A 2015 government report revealed that 34 million people (27% of the Japanese population) were aged 65 years and older, and 16 million people (13% of the Japanese population) were aged 75 years and older in 2015. According to projections, these proportions may reach 38% and 25% by 2055, respectively [1]. Longitudinal cohort studies in both Japan [2,3] and other Asian countries [4] have shown that the incident rate of functional decline or disability in activities of daily living (ADL) was higher in old-old (aged 75 years and older) than young-old (aged 65–74 years) populations. With the rapid increase in aged populations, the improvement of both quality and quantity of life has received increased research attention.

In general, functional capacity in older adults is divided into basic ADL (BADL) and instrumental ADL (IADL) [5]. BADL includes self-maintenance skills such as dressing, eating, bathing, and toileting, whereas IADL includes goal-oriented skills that are related to more complex and higher functional abilities, such as shopping, meal preparation, managing money [6,7]. Impairment in IADL precedes impairment in BADL [8,9]; therefore, a validated and culturally appropriate scale for measuring IADL is required to accurately detect individuals’ risk of functional disability.

With regard to IADL assessments, Lawton’s IADL scale was developed by Lawton and Brody in 1969 [7], and it has been widely used globally. Lawton’s IADL scale contains eight IADL items and has well-established reliability and validity [7]. In addition, Holbrook and Skilbeck (1983) developed the Frenchay Activities Index [10], which comprises 15 IADL items and has been deemed reliable and valid [11]. Further, Nouri and Lincoln (1987) proposed the Nottingham Extended Activities of Daily Living scale [12], which contains 22 IADL items grouped into four categories (mobility, kitchen, domestic, and leisure) and is also a reliable [12] and valid [13] assessment scale. However, demographic and economic change has had an effect on family structure and individuals’ lifestyle in recent decades. For example, the use of mobile phones, computers, and household appliances is part of everyday life [14]; however, the operation capability of electrical appliances was not assessed in the above-mentioned scales. Therefore, the IADL scale should be adapted to the recent living environment. Additionally, in Japan, the Tokyo Metropolitan Institute of Gerontology Index of Competence (TMIG-IC), containing 13 items related to IADL, was developed in 1991 to assess the IADL of community-dwelling older adults [9]. However, the TMIG-IC focuses on the assessment of higher-level competence including instrumental self-maintenance, intellectual activity, and social role [9]. Therefore, it does not cover the assessment of lower-level (relatively basic) IADL, which is of interest in the present study.

Therefore, we developed the National Center for Geriatrics and Gerontology Activities of Daily Living (NCGG-ADL) scale [15], comprising a 13-item self-report questionnaire regarding individuals’ ability to conduct IADL tasks that allow for discrimination of a wide range of IADL levels that correspond to a recent lifestyle, including the operation of electrical appliances. The NCGG-ADL is simple and highly reliable (Cronbach’s α = 0.937) [15], and a cut-off point to identify personal support or care needs in BADL was calculated as 12/13 points from previous cross-sectional data [15]. However, the predictive validity of the NCGG-ADL for detecting new incidences of personal support or care needs has not been sufficiently examined using longitudinal data.

Accordingly, we examined the predictive validity of IADL ability as measured by the NCGG-ADL scale as a means of detecting new incidences of functional disability among community-dwelling Japanese adults aged 75 years and older in a 24 month prospective cohort study. We hypothesized that the NCGG-ADL scale is an important predictor of the incidence of functional disability.

## 2. Materials and Methods 

### 2.1. Participants

This prospective cohort study involved community-dwelling older adults enrolled from a subcohort of the National Center for Geriatrics and Gerontology Study of Geriatric Syndromes (NCGG-SGS). NCGG-SGS is a Japanese national cohort study, the primary goal of which was to establish a screening system for geriatric syndromes and to validate evidence-based interventions for preventing them [16]. Overall, 2912 individuals aged ≥75 years completed our baseline assessment in the subcohort. All baseline assessments were carried out as health check-ups by well-trained study assistants in community centers. All staff received training from the authors on the protocols for administering the assessments before the study began. Inclusion criteria required that participants were aged ≥75 years and living in Nagoya or Obu city, Japan, at the time of examination (from June to December 2013).

This study included participants who completed baseline assessments, including an IADL measurement and follow-up assessment of disability by the national long-term care insurance (LTCI) system. We excluded participants based on the following criteria: 1) presence of disability based on the LTCI system at baseline (*n* = 35); 2) history of dementia (*n* = 16); 3) Mini-Mental State Examination (MMSE) score < 20 (*n* = 53); 4) death or relocation to another city during the follow-up period (*n* = 36); and 5) missing data concerning NCGG-ADL score or the above-described variables (*n* = 64). After exclusion, 2708 participants were followed up for 24 months and included in the final analysis of the present study.

The study protocol was developed in accordance with the Helsinki Declaration and was approved by the ethics committee of the National Center for Geriatrics and Gerontology (No. 637-3). Prior to study participation, written informed consent was obtained from all participants.

### 2.2. Assessment of Functional Disability

Participants were followed monthly as part of incident certification for personal support or care in the LTCI system during the 24 month period. Every Japanese national aged 65 years and older is eligible for benefits (in the form of institutional and community-based services, but not monetary support), based strictly on functional (physical and mental) disability. The nationally uniform criteria for long-term care need certification was objectively established by the Japanese government, and the computer-aided standardized needs-assessment system categorizes people into seven need levels [17].

The process for certification of personal support or care in the LTCI system is as follows: (1) an elderly person or caregiver contacts the municipal government to request official certification of the care needs of the applicant; (2) a trained local government official visits individuals’ homes to evaluate support or need for nursing care based on their current physical and mental status; (3) after completion of the assessment, the results are inputted into a computer to calculate the standardized scores on physical and mental status and the estimated time required for care in nine categories (grooming, bathing, eating, toileting, transferring, assistance with IADL, behavioral problems, rehabilitation, and medical services), and a care needs level based on the total estimated time for care is assigned; (4) the care needs certification board, which includes physicians, nurses, and other health and social services experts, reviews the data; and (5) the applicant is assigned to the level of care as required (certified support-level ranging from 1–2 or care-level ranging from 1–5). The eligibility of the individual receiving care via the LTCI system is re-evaluated every six months. In the present study, we received the certification data of care needs from the municipal government monthly, and incidences of functional disability were defined as a new certification of the LTCI service at any level.

### 2.3. Assessment of IADL

Self-reported ability in IADL was measured using the NCGG-ADL scale at baseline. The NCGG-ADL scale contains questions about 13 daily activities: (1) cut toenails, (2) go out by oneself, (3) take a bus or train, (4) shop for necessities, (5) transfer money, (6) look up a telephone number, (7) vacuum, (8) manage money, (9) control medications, (10) manage a house key, (11) cook, (12) use a microwave, and (13) use a gas stove. Participants reported their ability to conduct each of the 13 activities independently over the past month, using a simple dichotomous rating (yes/no). The score was calculated by summing the number of yes responses (0–13), with higher scores indicating higher ability in IADL [15].

### 2.4. Potential Confounding Factors

As covariates, sociodemographic variables (age, sex, body mass index, education, and presence of chronic diseases, including hypertension, diabetes mellitus, heart disease, Parkinson’s disease, stroke, and depression) were assessed using face-to-face interviews conducted by well-trained nurses at baseline. We also included the following covariates related to functional disability, based on previous study reports: living arrangements [18], fall history [19], and global cognitive function at baseline [20]. Current living arrangements (living alone or cohabiting) and fall history within the past year (at least one fall or no falls) were assessed by face-to-face interviews. Global cognitive function was measured using the MMSE; scores ranged from 0 to 30, with higher scores indicating better cognitive performance [21]. Participants who scored <28 points on the MMSE were considered to have mild cognitive impairment [22].

### 2.5. Statistical Analyses

Baseline characteristics were compared between participants who developed disabilities and those who remained independent using the Student’s t-test or Mann–Whitney U test for continuous variables and the χ^2^ test for categorical variables.

We calculated the survival rate of the incidence of functional disability during follow up according to cut-off points (12/13 points) [15] of total scores in the NCGG-ADL at baseline using Kaplan–Meier curves. Intergroup differences were estimated by the log-rank test. Cox proportional hazards regression models were used to analyze associations between ability in IADL as measured by the NCGG-ADL and the incidence of functional disability after adjusting for covariates.

All analyses were performed using SPSS Statistics 22 (IBM, Tokyo, Japan). Significance was set at *p* < 0.05.

### 2.6. Patients and Public Involvement

Patients or the public were not involved in the designing or planning of the study.

## 3. Results

### 3.1. Characteristics of Participants with and without Functional Disability

Of the 2708 participants, 430 (15.9%) showed IADL limitations (≤12 points on the NCGG-ADL scale) at baseline, and 289 (10.7%) participants developed functional disability during the 24 month follow-up period. Compared with those who remained independent, participants who developed disability were significantly older, less educated, more likely to have heart disease, to live alone, to have a fall history, have lower MMSE scores, and more likely to have mild cognitive impairment (<28 points on the MMSE). With regard to IADL measured by the NCGG-ADL scale, those who developed disability had a significantly lower NCGG-ADL score and were more likely to have an IADL limitation (≤12 points on the NCGG-ADL scale) than those who remained independent (Table 1). 

### 3.2. Association between Ability in IADL as Measured by the NCGG-ADL and the Incidence of Functional Disability

**Figure 1:**
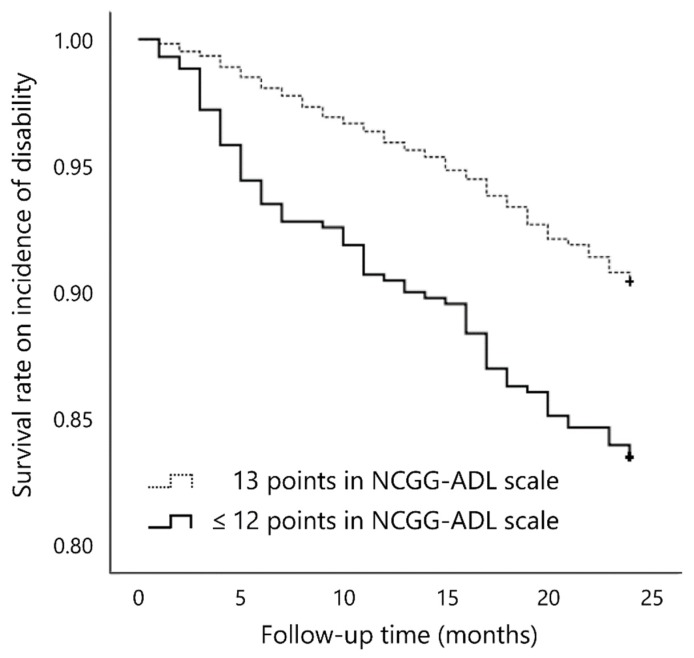
Estimates of survival rate on disability incidence according to score on the NCGG-ADL scale at baseline.

In the Kaplan–Meier log-rank test, participants who scored ≤12 points on the NCGG-ADL at baseline had a significantly higher risk of disability incidence than did those who scored 13 points (*p* < 0.001; Figure 1). 

Cox regression analysis showed that the hazard ratio (HR) and 95% confidence interval (CI) for new incidences of functional disability per point of the NCGG-ADL scale was 0.79 (95%CI: 0.73–0.86, *p* < 0.001) in the crude model and 0.84 (95%CI: 0.77–0.92, *p* < 0.001) in the adjusted model including covariates (age, sex, body mass index, education, hypertension, diabetes mellitus, heart disease, Parkinson’s disease, stroke, depression, living alone, fall history, and mild cognitive impairment; Table 2).

In addition, HR and 95%CI for new incidences of functional disability in participants who scored ≤12 points were 1.82 (95%CI: 1.39–2.38, *p* < 0.001) in the crude model and 1.58 (95%CI: 1.19–2.09, *p* = 0.002) in the adjusted model including covariates (age, sex, body mass index, education, hypertension, diabetes mellitus, heart disease, Parkinson’s disease, stroke, depression, living alone, fall history, and mild cognitive impairment; Table 3). 

To examine the robustness of the association between IADL, measured by the NCGG-ADL scale, and the incidence of functional disability, we conducted a sensitivity analysis by excluding respondents who developed functional disability within 3 months (*n* = 27). The HR and 95%CI for the new incidences of functional disability in participants who scored ≤12 points was 1.63 (95%CI: 1.22–2.18, *p* = 0.001) in the crude model and 1.41 (95%CI: 1.04–1.92, *p* = 0.026) in the adjusted model (Table S1). The finding of the sensitivity analysis was consistent with our main analysis.

## 4. Discussion

The results of this prospective cohort study showed that limitation in IADL was significantly associated with the incidence of functional disability over 24 months after adjusting for covariates. Ability in IADL, as measured by the NCGG-ADL scale, is thought to be useful for detecting new incidences of disability among community-dwelling Japanese aged ≥75 years.

Previous studies have shown that the prevalence of IADL limitation ranged from 19% to 32% among elderly aged ≥65 years [23,24,25]. In general, the probabilities of IADL disability increase with age, and thus our prevalence of IADL limitation was slightly lower than that observed in previous studies. We excluded participants who already had BADL disability at baseline, which might have led to the underestimation of the prevalence of IADL limitation. In other words, the prevalence of IADL limitation reached 15.9% even though participants had no prior BADL disability, which suggests that impairment in IADL precedes impairment in BADL, as reported in previous studies [8,9].

Previous studies have reported that the incident rate of disability over two years ranged from 8% to 17% among community-dwelling adults aged ≥75 years [26,27,28], which is consistent with the current results. At baseline, participants’ age, education, the proportion of having heart disease and fall history, the proportion of living alone, and global cognitive function were significantly different depending on whether they developed functional disability during follow up. These results are also in line with previous studies that examined the effects of age [29], education level [29], heart disease [29], living arrangement [18], fall history [19], and global cognitive function [20] on community-dwelling older adults’ functional disability. In particular, mild cognitive impairment (<28 points on the MMSE) was found to be associated with functional disability even after conducting a multivariable analysis using Cox regression. A previous study reported that cognitive impairment (but not dementia) was associated with future functional disability, and the authors argued that they may interact synergistically [20]. Further longitudinal research is required to identify the temporal relationship between cognitive impairment and functional disability. On the other hand, morbidities (hypertension, diabetes mellitus, heart disease, Parkinson’s disease, stroke, and depression) did not have a significant impact on functional disability in our multivariable analysis. The medical conditions were assessed by participants’ self-report in our study, and our participants might be physically fit enough to visit the health check-up site. This could be the reason that the relationship between morbidities and functional disability was not significant in the present study.

Concerning the relationship between IADL and functional disability, IADL limitation was significantly associated with new incidences of disability during the 24 month follow up after adjusting for covariates. Our results from prospective data showed that the NCGG-ADL scale has predictive validity for detecting incidences of functional disability among community-dwelling Japanese aged ≥75 years. Previous studies reported that IADL ability is regarded as hierarchically superior to that of BADL [30,31], and IADL disability is generally more sensitive than BADL disability to contextual changes [32]. Therefore, early detection of IADL disability is critical to predict and prevent BADL disability.

The present study has several strengths and implications. The NCGG-ADL scale is a simplified and rapid questionnaire based on self-reported IADL ability that is highly reliable [15]. Therefore, the scale is appropriate as a primary screening tool that can be widely conducted without medical expertise. In addition, we included a relatively large sample of cohort data, including monthly follow up of functional disability based on the national LTCI system. Our findings firmly reinforce recent findings elucidating the relationship between IADL limitation and functional disability. Although our results may not be generalizable to the entire older population (including those aged 65–74 years), our findings are essential for developing tailored prevention strategies for this specific high-risk population (i.e., those aged ≥75 years).

This study also has some limitations. First, the average score on the NCGG-ADL was 12.7 points out of 13. The result indicates a possible ceiling effect, which may have increased the possibility of beta error and reduced the statistical power of our analysis. Moreover, we could not use the score on the NCGG-ADL scale as a continuous variable in our final analysis because of biased distribution (ceiling effect). Further investigation is required to examine the predictive validity of the raw score on the NCGG-ADL scale for detection of functional disability incidence or compare the weight of effect on functional disability among the IADL items. Second, we conducted health check-ups at community centers; therefore, participants might have been relatively more health conscious and physically active than those who could not visit the site of check-up. Therefore, selection bias should be taken into consideration when interpreting our findings. Third, previous studies demonstrated that IADL status was influenced by race [33], residential area [25], and socioeconomic factors such as marital status and occupational type [25]. Therefore, further research examining its cross-cultural validity using diverse populations is required.

## 5. Conclusions

In conclusion, our findings indicate that IADL limitation as measured by the NCGG-ADL scale could be a useful predictor of new incidences of functional disability. The NCGG-ADL was validated to screen for functional disability among community-dwelling Japanese adults aged ≥75 years. Early detection of IADL disability is vital to predict and prevent functional disability.

## Figures and Tables

**Table 1:** Baseline characteristics of participants (*N* = 2708).

	Overall (*n* = 2708) *M* (*SD*)	Independent (*n* = 2419) *M* (*SD*)	Incident Disability (*n* = 289) *M* (*SD*)	*p*
Age (years)	79.0 (3.5)	78.8 (3.3)	81.1 (3.9)	<0.001
Body mass index (kg/m^2^)	22.9 (3.0)	23.0 (3.0)	22.6 (3.3)	0.053
Education (years)	11.7 (2.7)	11.8 (2.7)	11.4 (2.6)	0.028
MMSE (score)	25.7 (2.5)	25.8 (2.4)	25.0 (2.6)	<0.001
NCGG-ADL (score)	12.7 (0.8)	12.8 (0.7)	12.5 (1.3)	<0.001
	***n* (%)**	***n* (%)**	***n* (%)**	***p***
Female	1396 (51.6)	1234 (51.0)	162 (56.1)	0.105
Medical conditions:				
Hypertension	1472 (54.4)	1307 (54.1)	165 (57.1)	0.327
Diabetes mellitus	361 (13.3)	316 (13.1)	45 (15.6)	0.238
Heart disease	579 (21.4)	503 (20.9)	76 (26.5)	0.028
Parkinson’s disease	11 (0.4)	9 (0.4)	2 (0.7)	0.419
Stroke	190 (7.0)	165 (6.8)	25 (8.7)	0.254
Depression	103 (3.8)	92 (3.8)	11 (3.8)	0.999
Living alone	476 (17.6)	413 (17.1)	63 (21.9)	0.044
Fall history	593 (21.9)	510 (21.1)	83 (28.7)	0.003
Mild cognitive impairment (<28 points on the MMSE)	1973 (72.9)	1737 (71.8)	236 (81.7)	<0.001
IADL limitation (≤12 points on the NCGG-ADL scale)	430 (15.9)	359 (14.8)	71 (24.6)	<0.001

M, mean; SD, standard deviation; MMSE, Mini-Mental State Examination; IADL, instrumental activities of daily living; NCGG-ADL, the National Center for Geriatrics and Gerontology Activities of Daily Living scale.

**Table 2:** Hazard ratios and 95% confidence intervals for disability incidence in the crude and adjusted models for 24 months per the raw NCGG-ADL score (*N* = 2708).

		Crude Model			Adjusted Model		
		HR	95% CI	*p*	HR	95% CI	*p*
NCGG-ADL score	(points)	0.79	0.73–0.86	<0.001	0.84	0.77–0.92	<0.001
Age	(years)				1.14	1.11–1.17	<0.001
Female	(yes)				1.39	1.07–1.80	0.014
Body mass index	(kg/m^2^)				0.97	0.94–1.01	0.179
Education	(years)				1.01	0.97–1.06	0.632
Hypertension	(yes)				1.04	0.82–1.33	0.724
Diabetes mellitus	(yes)				1.21	0.87–1.67	0.259
Heart disease	(yes)				1.31	1.00–1.71	0.052
Parkinson’s disease	(yes)				1.75	0.43–7.07	0.435
Stroke	(yes)				1.15	0.76–1.75	0.503
Depression	(yes)				0.98	0.54–1.80	0.952
Living alone	(*n*, %)				0.82	0.61–1.11	0.199
Fall history	(*n*, %)				1.28	0.98–1.66	0.066
Mild cognitive impairment	(*n*, %)				1.53	1.12–2.08	0.008

HR, hazard ratio; CI, confidence interval; IADL, instrumental activities of daily living; NCGG-ADL, the National Center for Geriatrics and Gerontology Activities of Daily Living scale.

**Table 3:** Hazard ratios and 95% confidence intervals for disability incidence in the crude and adjusted models for 24 months per the cut-off points (12/13 points) of the NCGG-ADL scale (*N* = 2708).

		Crude Model			Adjusted Model		
		HR	95% CI	*p*	HR	95% CI	*p*
NCGG-ADL scale							
13 points		Reference			Reference		
≤12 points		1.82	1.39–2.38	<0.001	1.58	1.19–2.09	0.002
Age	(years)				1.14	1.11–1.18	<0.001
Female	(yes)				1.42	1.10–1.85	0.008
Body mass index	(kg/m^2^)				0.97	0.93–1.01	0.150
Education	(years)				1.01	0.97–1.06	0.573
Hypertension	(yes)				1.06	0.83–1.35	0.667
Diabetes mellitus	(yes)				1.24	0.90–1.71	0.197
Heart disease	(yes)				1.29	0.99–1.69	0.061
Parkinson’s disease	(yes)				1.73	0.43–7.01	0.442
Stroke	(yes)				1.17	0.78–1.78	0.447
Depression	(yes)				0.96	0.53–1.77	0.907
Living alone	(*n*, %)				0.82	0.61–1.11	0.201
Fall history	(*n*, %)				1.26	0.97–1.64	0.081
Mild cognitive impairment	(*n*, %)				1.54	1.12–2.10	0.007

HR, hazard ratio; CI, confidence interval; IADL, instrumental activities of daily living; NCGG-ADL, the National Center for Geriatrics and Gerontology Activities of Daily Living scale.

## Supplementary Materials

The following are available online at https://www.mdpi.com/1660-4601/17/7/2291/s1, Table S1: Results of the sensitivity analysis examining the association between IADL measured by the NCGG-ADL scale and functional disability incidence (*N* = 2681).
