# Supplementary material for: Predictive Validity of a New Instrumental Activities of Daily Living Scale for Detecting the Incidence of Functional Disability among Community-Dwelling Older Japanese Adults: A Prospective Cohort Study
Source: Int J Environ Res Public Health. 2020 Mar 29;17(7):2291. doi: 10.3390/ijerph17072291 (PMC7177260; doi:10.3390/ijerph17072291)
Supplement: Supplementary file 1 [file ijerph-17-02291-s001.pdf]

Table S1. Results of the sensitivity analysis examining the association between IADL measured by the NCGG-ADL scale and functional disability incidence ($N = 2681$).

		Crude Model			Adjusted Model		
		**HR**	**95% CI**	***P***	**HR**	**95% CI**	***P***
NCGG-ADL scale:							
13 points			Reference			Reference	
≤ 12 points		1.63	1.22–2.18	0.001	1.41	1.04–1.92	0.026
Age	(years)				1.13	1.09–1.16	<0.001
Female	(yes)				1.40	1.06–1.84	0.016
Body mass index	(kg/m^2)				0.97	0.93–1.01	0.130
Education	(years)				1.01	0.96–1.06	0.657
Hypertension	(yes)				1.08	0.84–1.40	0.559
Diabetes mellitus	(yes)				1.24	0.88–1.74	0.222
Heart disease	(yes)				1.27	0.96–1.69	0.098
Parkinson's disease	(yes)				1.90	0.47–7.70	0.370
Stroke	(yes)				1.19	0.77–1.83	0.440
Depression	(yes)				0.95	0.51–1.79	0.877
Living alone	(*n*, %)				0.87	0.64–1.20	0.396
Fall history	(*n*, %)				1.30	0.99–1.71	0.060
Mild cognitive impairment	(*n*, %)				1.68	1.21–2.35	0.002

HR, hazard ratio; CI, confidence interval; IADL, instrumental activities of daily living; NCGG-ADL, the National Center for Geriatrics and Gerontology Activities of Daily Living scale.
